# Differential depth of treatment response required for optimal outcome in patients with blast phase versus chronic phase of chronic myeloid leukemia

**DOI:** 10.1038/bcj.2017.4

**Published:** 2017-02-03

**Authors:** Z Chen, L J Medeiros, H M Kantajian, L Zheng, Z Gong, K P Patel, H Xiong, W Wang, J E Cortes, S Hu

**Affiliations:** 1 Department of Hematopathology, The University of Texas MD Anderson Cancer Center, Houston, TX, USA; 2 Department of Leukemia, The University of Texas MD Anderson Cancer Center, Houston, TX, USA; 3 Department of Hematology, Shanghai Xuhui Central Hospital, Shanghai, China

The outcome of patients with chronic myeloid leukemia (CML) has improved dramatically following the introduction of tyrosine kinase inhibitor (TKI) therapy.^1^ With TKI treatment, most patients with CML, chronic phase (CML-CP) can achieve complete cytogenetic response (CCyR) within 12 months after initial diagnosis of CML. Thanks to the high sensitivity of molecular methods to detect *BCR-ABL1* transcripts, deeper molecular responses can now be ascertained, including major molecular response (MMR), MR4 or MR4.5 (4 and 4.5- log reduction of *BCR-ABL1* fusion transcripts, respectively), and molecularly undetectable leukemia (MUL).^2, 3^ It seems intuitive that the deeper the treatment response, the better the survival of patients with CML-CP is.^4, 5^ However, multiple studies have shown that deeper responses beyond CCyR confer no survival benefit. Patients with CML-CP who achieve CCyR have a very favorable survival similar to that of the general population.^6, 7^ In contrast, CML-CP patients who are unable to achieve CCyR have a significantly poorer survival, suggesting that achieving CCyR is essential for predicting a favorable outcome.

In the pre-TKI era, in most patients the disease progressed to blast phase (BP) within 3–4 years after initial diagnosis of CML-CP. With TKI therapy, the progression of CML from CP to BP has decreased substantially and the long-term cumulative probability of such progression is currently about 5%.^1, 8^ Despite the revolutionary progress in the treatment of patients with CML-CP, CML-BP remains a therapeutic challenge. In general, CML-BP is a fatal disease in the era of TKI therapy, with a median survival of only 6–10 months.^9, 10^ Further investigation into the potential relationship between the depth of treatment response and optimal patient survival is warranted. Here we investigate a large cohort of patients with CML-BP treated in the era of TKIs and determine whether achieving CCyR is adequate for optimal survival or if deeper molecular responses have a prognostic value.

Cases of CML-BP that met following selection criteria were included in this study: 1, CML-BP diagnosed from 2001 to 2016; 2, presence of t(9;22)(q34;q11.2) or variant translocations detected by conventional karyotyping analysis; and 3, available data regarding molecular response beyond CCyR if achieved. Patients who presented with isolated myeloid sarcoma without concurrent BP in the bone marrow or peripheral blood and patients with *BCR-ABL1*-positive *de novo* acute leukemia were excluded in the study. The blast phase was defined as 30% or more blasts in the bone marrow or peripheral blood. Overall survival (OS) was calculated from the date of diagnosis of BP to the date of last follow-up or death. The study was approved by the Institutional Review Board of the University of Texas MD Anderson Cancer Center.

In total, 386 patients with CML-BP were included in this study, including 253 (65.5%) patients with myeloid BP (MyBP), 121 (31.3%) patients with lymphoid BP (LyBP) and 12 (3.1%) patients with mixed-phenotype BP. There were 252 (65.3%) men and 134 (34.7%) women with a median age of 51.9 years (range: 13.2–90.2 years) at the time of diagnosis of CML-BP. The median interval time from initial diagnosis of CML to onset of BP was 22.5 months (range: 0–238.5 months). After onset of BP, 361 (93.5%) patients received TKI treatment, 311 (80.6%) received chemotherapy and 116 (30.1%) underwent allogeneic stem cell transplantation. The clinical characteristics of the entire cohort and each subgroup are listed in Table 1.

These patients were further stratified into five subgroups based on the depth of treatment response: 1, no hematologic response (HR), which included 141 patients; 2, HR only, which included 94 patients who achieved HR but not CCyR; 3, CCyR only, which included 31 patients who achieved CCyR but not MMR; 4, MMR, which included 28 patients who achieved MMR but not molecularly undetectable leukemia (MUL); and 5, MUL, which included 92 patients (Table 1). Patients who achieved deeper responses beyond MMR but not MUL were lumped with MMR due to low numbers of patients and the test results falling beyond the linearity of our assay once the *BCR-ABL1*:*ABL1* ratio was <0.01%.

The OS of patients with CML-BP was poor (Figure 1a). Patients with myeloid BP had a worse survival than those with lymphoid BP (*P*=0.0004). The median OS was 9.2 months for patients with MyBP, 19.2 months for those with LyBP and 11.4 months for the entire cohort.

We first analyzed the relationship between the depth of treatment response and patient survival of the five subgroups in the entire cohort. There was a significant difference in survival among the five subgroups of patients (Figures 1b, *P*<0.0001), correlating with the depth of remission and a general trend between a deeper response and a longer survival. As expected, patients without HR had the worst outcome with a median OS of 3.6 months. In contrast, patients with MUL had the best outcome with a median survival of 132.2 months and 5-year OS of 71.8%. Patients with MMR, CCyR and HR had an intermediate outcome with a median survival of 38.0, 17.6 and 11.0 months, and 5-year OS was 34.4, 12.2 and 11.0%, respectively. There was no significant difference in survival between patients who achieved CCyR and those who achieved HR (*P*=0.33).

We then analyzed the relationship between the depth of response and survival in CML patients with MyBP or LyBP separately. As shown in Figures 1c and d, there was a general trend between a better survival and a deeper molecular response in both MyBP and LyBP groups. In both groups, patients who achieved HR had significantly better OS than those who did not achieve HR (*P*<0.0001 for both groups), and patients who achieved MUL had a better survival than those who achieved MMR (*P*=0.11 for the MyBP group and *P*=0.037 for the LyBP group). There was no significant difference between patients who achieved CCyR and those who achieved HR in neither groups. The lack of statistical significance between some of these subgroups pairwise was related, at least in part, to the low numbers of patients, particularly low numbers of patients who achieved CCyR or MMR.

Of note, 80 of 92 (87.0%) CML-BP patients who achieved MUL received allogeneic hematopoietic stem cell transplantation (Table 1), re-enforcing the notion that allogeneic hematopoietic stem cell transplantation is an excellent treatment option for patients with CML-BP. The other 12 CML patients, including eight patients with MyBP and four patients with LyBP, achieved MUL following combined chemotherapy plus TKIs. These patients had a similar survival to the patients who achieved MUL after allogeneic hematopoietic stem cell transplantation (median OS: 132.2 months, *P*=0.61).

The data presented here show that the impact of depth of treatment response on survival is different in patients with CML-CP versus patients with CML-BP. Patients with CML-CP who achieve CCyR have a survival more favorable than those who achieve only HR, and their survival is similar to those who achieve MMR and the general population.^6, 7^ Therefore, achieving MMR, not MUL, is the primary goal of therapy. In contrast, patients with CML-BP who achieve CCyR have a dismal 5-year survival rate similar to those who achieve HR and significantly worse than those who achieve MMR. CML-BP patients who achieve MMR also have a poor 5-year survival rate (34.4% in this study). By contrast, patients with CML-BP who achieve MUL have a much better outcome with a 5-year survival rate of 71.8% and a median OS of 132.2 months in this study.

In an earlier study, Hehlmann *et al.*^11^^,12^ reported a more favorable survival in CML-CP patients who achieved a deeper molecular response, although MR4.5 versus CCyR had a smaller effect on survival than CCyR versus no CCyR, given the superior survival of those who achieved CCyR. Despite the smaller survival benefit, achieving MR4.5 is clinically meaningful as it may serve as an indicator of a step toward facilitating successful discontinuation of treatment.^12, 13^ In contrast, CML-BP patients who achieve CCyR have a dismal outcome and the survival advantage of deeper molecular responses (MUL and MMR) versus CCyR is easily appreciated. Thus, it seems true that a deeper response to therapy correlates with a longer survival in both CML-CP and CML-BP patients, albeit to a different degree and with different clinical implications. Interestingly, the outcome of CML-BP patients who achieve CCyR, MMR and MUL somewhat mirrors that of *de novo* acute myeloid leukemia patients under 60 years who have poor, intermediate and favorable cytogenetics, respectively.^14, 15^ However, due to low numbers of patients who achieve MMR or deeper response but not MUL and technical limitation in our assay, we cannot accurately determine the relationship between patient survival and MR4 or MR4.5.

In conclusion, a deeper molecular response correlates with a better survival in patients with CML, blast phase. However, achieving CCyR and MMR is inadequate for the optimal survival, and achieving molecularly undetectable leukemia might be the ultimate goal for excellent outcome in patients with CML, blast phase. Our study results could provide a basis for a guideline in the management of CML-BP different from that in the management of CML-CP.

## Figures and Tables

**Figure 1:**
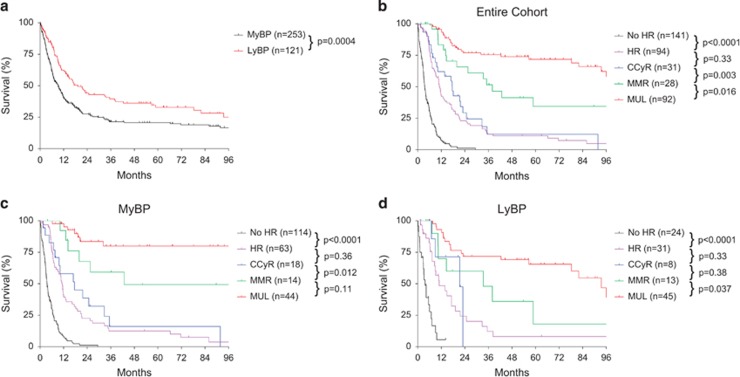
Impact of depth of treatment response on survival in patients with CML, blast phase. (**a**) Overall survival of the entire cohort of patients with CML, MyBP and LyBP. (**b**) Depth of treatment response-dependent survival in the entire cohort of patients with CML-BP. (**c**) Depth of treatment response-dependent survival in patients with CML, MyBP. (**d**) Depth of treatment response-dependent survival in patients with CML, LyBP. BP, blast phase; CML, chronic myeloid leukemia; LyBP, lymphoid blast phase; MyBP, myeloid blast phase.

**Table 1:** Clinical characteristics of 386 patients with chronic myeloid leukemia, blast phase in the era of tyrosine kinase inhibitor therapy

			
	*All BP*^a^ *(*n=*386)*					*MyBP (*n=*253)*					*LyBP (*n=*121)*				
	*No HR*	*HR*	*CCyR*	*MMR*	*MUL*	*No HR*	*HR*	*CCyR*	*MMR*	*MUL*	*No HR*	*HR*	*CCyR*	*MMR*	*MUL*
Number	141	94	31	28	92	114	63	18	14	44	24	31	8	13	45
															
*Sex, n (%)*															
Male	94 (66.7)	57 (60.6)	18 (58.1)	21 (75.0)	62 (67.4)	75 (65.8)	36 (57.1)	8 (44.4)	11 (78.6)	29 (65.9)	18 (75.0)	21 (67.7)	5 (62.5)	9 (69.2)	32 (71.1)
Female	47 (33.3)	37 (39.4)	13 (41.9)	7 (25.0)	30 (32.6)	39 (34.2)	27 (42.9)	10 (56.6)	3 (21.4)	15 (34.1)	6 (25.0)	10 (32.3)	3 (37.5)	4 (30.8)	13 (28.9)
															
*Age (years)*															
Median	56.0	56,5	47.1	48.8	48.2	55.3	55.6	47.3	48.7	51.0	56.6	60.5	50.0	48.1	45.4
Range	15.4–90.2	13.2–79.4	27.0–81.3	22.4–77.0	13.6–75.5	15.4–90.2	24.9–79.4	31.0–74.6	25.3–61.2	25.6–72.1	27.3–73.3	13.2–75.9	27.0–81.3	22.4–77.0	13.6–75.5
															
*Interval (months)*															
Median	30.9	25.5	23.4	16.7	9.3	34.0	28.9	15.3	16.0	9.5	12.7	12.0	38.3	17.2	9.3
Range	0.0–232.1	0.0–238.5	0.0–125.5	0.0–225.5	0.0–228.7	0.0–232.1	0.0–238.5	0.0–125.5	0–156.6	0–202.0	0.0–218.1	0.0–101.5	0.0–106.2	0.0–225.5	0.0–228.7
															
*Status at last F/U, n (%)*															
Alive	10 (7.1)	18 (19.1)	8 (25.8)	14 (50.0)	64 (69.6)	6 (5.3)	11 (17.5)	4 (22.2)	8 (57.1)	35 (79.5)	4 (16.7)	7 (22.6)	4 (50.0)	6 (46.2)	27 (60.0)
Dead	131 (92.9)	76 (81.9)	23 (74.2)	14 (50.0)	28 (30.4)	108 (94.7)	52 (82z.5)	14 (77.8)	6 (42.9)	9 (20.5)	20 (83.3)	24 (77.4)	4 (50.0)	7 (53.8)	18 (40.0)
															
*Treatment, n (%)*															
With TKI	123 (87.2)	92 (97.9)	29 (93.5)	27 (96.4)	90 (97.8)	97 (85.1)	61 (96.8)	17 (94.4)	13 (92.9)	43 (97.7)	23 (95.8)	31 (100.0)	7 (87.5)	13 (100)	44 (97.8)
Without TKI	18 (12.8)	2 (2.1)	2 (6.5)	1 (3.2)	2 (2.2)	17 (14.9)	2 (3.2)	1 (5.6)	1 (7.1)	1 (2.3)	1(4.2)	0 (0.0)	1 (12.5)	0 (0.0)	1 (2.2)
Allo-HSCT	2 (1.4)	14 (14.9)	9 (29.0)	11 (39.3)	80 (95.7)	1 (0.9)	11 (17.5)	7 (38.8)	7 (50.0)	36 (81.2)	1 (4.2)	3 (9.7)	1 (12.5)	3 (23.1)	41 (91.1)

Abbreviations: Age, age at diagnosis of BP; Allo-HSCT, allogeneic hematopoietic stem cell transplantation; BP, blast phase; CCyR, complete cytogenetic response but no MMR; F/U, follow-up; HR, hematologic response but no CCyR; Interval, interval time from initial diagnosis of CML to the diagnosis of BP; LyBP, lymphoid BP; MMR, major molecular response or deeper but no MUL; MUL, molecularly undetectable leukemia; MyBP, myeloid BP; TKI, tyrosine kinase inhibitor; *n*, number.

a Include 12 cases of CML-BP with mixed phenotype. Hematologic response is defined as <5% blasts in bone marrow and 0% blasts in peripheral blood. CCyR, MMR and MUL are defined according to the NCCN and European LeukemiaNet Guidelines.
